# Environmentally safe (chlorine-free): new green propellant formulation based on 2,2,2-trinitroethyl-formate and HTPB†

**DOI:** 10.1039/c8ra01515e

**Published:** 2018-03-27

**Authors:** Mohamed Abd-Elghany, Thomas M. Klapötke, Ahmed Elbeih

**Affiliations:** Department Chemie, Ludwig-Maximilians Universität München 81377 München Germany tmk@cup.uni-muenchen.de; Military Technical College Kobry Elkobbah Cairo Egypt

## Abstract

A new green (chlorine-free) high energy dense oxidizer (HEDO) 2,2,2-trinitroethyl-formate (TNEF) and its propellant formulation based on the hydroxyl-terminated polybutadiene (HTPB) as a binder was prepared and studied. The new oxidizer TNEF was successfully prepared and characterized by nuclear magnetic resonance (NMR) and FTIR spectrometer. Scanning electron microscopy (SEM) was used to check the crystal morphology of the oxidizer. A high specific impulse (*I*_s_ = 250.1 s) was obtained from the characteristics calculation of the new oxidizer instead of (*I*_s_ = 156.9 s) for the commonly used ammonium perchlorate (AP) by using EXPLO5_V6.03 software. The burning behavior and the burning rate were determined by using a high speed camera. TNEF and the propellant formulations were studied by using nonisothermal thermogravimetric analysis (TGA) and the kinetic parameters of the studied samples were determined by using isoconversional (model-free) methods “Kissinger, Ozawa and Flynn–Wall (OFW) and Kissinger–Akahira–Sunose (KAS)”. The results proved that the new oxidizer and its formulation based on HTPB have chlorine-free decomposition products and have higher performance characteristics than the traditional propellants.

## Abstract 2

Burning behavior of a traditional (AP/HTPB) and new (TNEF/HTPB) propellants.

## Graphical abstract

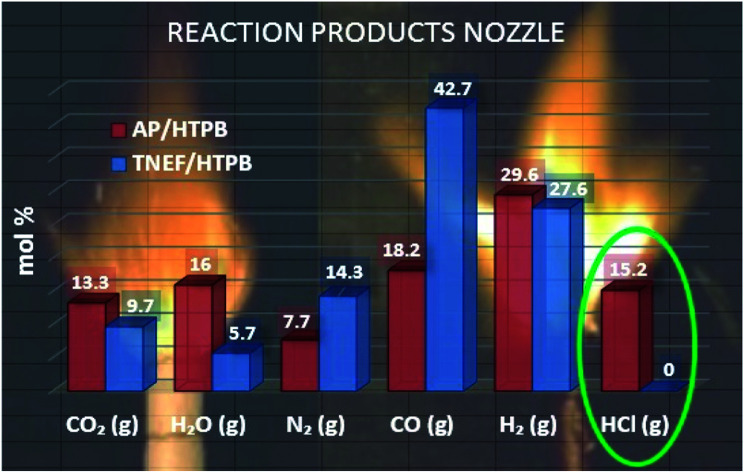

## Introduction

Due to the large merits of high compatibility, low viscosity and the superior mechanical properties, hydroxyl-terminated polybutadiene (HTPB) became one of the most commonly used polymeric binders in several fields specially in the field of composite solid rocket propellants.^1^ For the simplicity, reliability and lower propulsion system cost of the solid propellants, they have an immense range of applications in tactical rockets, submarine-based ballistic missiles, space launcher boosters and even amateur hobby rockets.^2,3^ These propellants are composed of a polymeric matrix that loaded with a solid powder oxidizer and possibly of a metal powder which plays the role of a secondary fuel component. Despite its smoke combustion and toxic gaseous products, still ammonium perchlorate (AP) is the most widely used oxidizer for composite solid propellants.^4–6^

Composite solid propellants that based on AP and HTPB are well known for their good performance characteristics and relatively low cost of manufacturing, but their limitations regarding toxicity and environmental impact are also well documented. Perchlorate contamination is becoming a more widespread concern in many countries all over the world.^7–12^ At high concentrations, perchlorate can affect thyroid gland functions. Away from influencing the thyroid activity in humans, AP produce large amount of hydrochloric acid (HCl) during its combustion. Future propellants should not have such major hazards that cause diverse harmful to the crew or ground handling personnel. Green propellant formulations (chlorine-free) would highly reduce the risks of toxicity, operational handling complexity, spacecraft contamination, and environmental contamination hazardous.

Many researchers are working on solving the toxicity problems of AP without affecting the propellant performance.^13^ To achieve this target, numerous researches have been studied based on adding some additives such as metals or nitramines (RDX, HMX, *etc.*).^14–18^ Several groups worldwide have intensively investigated other compounds to substitute AP to overcome its toxicity problems and to enhance the energetic characteristics, sensitivities and thermal properties.^19–21^ These compounds are based on orthocarbonates, tetrazoles, carbamates, nitro-carbamates, formates, pyrazoles and triazoles.^22–24^ Epishina *et al.*^25^ has synthesized 2,2,2-trinitroethanol (TNE) using Henry and Mannich reactions, which is important and suitable starting material for a numerous compounds which have been synthesized during the recent studies.^26^ 2,2,2-Trinitroethyl-formate (TNEF) is a new interesting high energy dense oxidizer (HEDO) that has density of 1.81 g cm^−3^, oxygen balance of (ΩCO_2_) of 10.1%, impact sensitivity of 5 J and friction sensitivity of 96 N.^27^

TNEF is a chlorine-free HEDO, which might have high performance and has not been studied in any propellant formulation yet. Moreover, studying of the thermal behavior and kinetics of reaction for the new energetic materials are essential to find suitable new applicable applications.^28–36^ In this paper, preparation and characterization of TNEF were presented. Propellant formulations based on HTPB as a binder and TNEF and AP as oxidizers have been prepared. EXPLO5 V_6.03 has been used to study the burning characteristics and decomposition products of the samples. The burning rate of the propellants was measured. The thermal behavior and decomposition kinetics of the individual HTPB and TNEF in addition to the two propellant formulations were studied using TGA technique. Different isoconversional methods for calculation were applied to determine the kinetic parameters of the HTPB, TNEF and the new propellant formulations.

## Experimental

### Materials

Hydroxyl-terminated polybutadiene (HTPB, R-45M of ARCO Co.) as a pre-polymer with a hydroxyl content of 0.84 meq g^−1^, hexamethylene diisocyanate (HMDI) as a curing agent with an NCO equivalence value of 11.83 meq g^−1^, chloroform, anhydrous iron(iii) chloride and diethyl ether, which were obtained from Sigma-Aldrich; 2,2,2-trinitroethanol (TNE) which was prepared in our laboratories (AK Klapötke).

#### Synthesis of 2,2,2-trinitroethyl-formate (TNEF)

The synthesis of the air- and moisture-sensitive materials were done in an inert atmosphere of dry nitrogen using Schlenk techniques.^37^ The chloroform was freshly distilled prior to use. 2,2,2-Trinitroethanol (10 g, 56.0 mmol) was dissolved in dry chloroform (20 mL), anhydrous iron(iii) chloride (0.8 g, 4.92 mmol) was added under careful exclusion of moisture. The mixture was heated under reflux at 85 °C for 5 days. After cooling, the content of reaction was poured into diethyl ether (100 mL). The ether solution was washed with cold water (3 × 100 mL) and dried over magnesium sulfate. After removing the solvent, a creamy coloured crude product was obtained, which was recrystallized from dichloromethane to yield 7.6 g (74% yield) of colorless crystals of 2,2,2-trinitroethyl-formate.

#### Preparation of the propellants formulation

The preparation process is based on mixing of the oxidizer (TNEF, 80 wt%) with the pre-polymer (HTPB) in a 200 mL vertical mixer for 40 minutes at 40 °C under vacuum to drive out entrapped air. Then, the curative (HMDI) was added at 55 °C. Mixing process remained for another 30 minutes. Finally, the prepared propellant samples were put in a specific mold and were cured in a vacuum oven at 60 ± 2 °C for seven days. The weight percentage of the binder system was 20 wt%. The AP/HTPB formulation was prepared by the same method.

#### Experimental techniques

The NMR spectra were recorded for TNEF by a JEOL Eclipse 400 instrument, and the chemical shifts were determined with respect to the external standards Me_4_Si (^1^H, 399.8 MHz; ^13^C, 100.5 MHz) and MeNO_2_ (^14^N, 28.8 MHz). Elemental analysis of C, H, N were performed with an Elementar Vario EL Analysis. The IR spectra were recorded at ambient temperature by a Perkin-Elmer Spectrum BX-FTIR spectrometer equipped with a Smiths DuraSamplIR II attenuated total reflectance (ATR) device. Determination of sensitivities to different stimuli was studied by BAM falling hammer test to determine the impact sensitivity (IS) according to STANAG 4489 (ref. 38) and BAM friction tester (ODG 632 GmbH) for the determination of the friction sensitivity (FS) according to STANAG 4487.^39^ EXPLO5 thermodynamic code version_6.03 has been used to determine the combustion characteristics of the propellant samples. The combustion conditions are based on the ideal gas equation of state with 70 atm combustion chamber pressure and under isobaric combustion. The specific impulse of the propellant samples were recorded. The burning rate of the studied propellants was measured by using a high-speed camera.^40^ Model (visario g2 1500) with frame measurements (1.000 fps). The propellant samples were prepared in the form of cylinders with dimension of 100 mm length and 8 mm diameter and the burning rate was measured at atmospheric pressure (0.1 MPa). Each sample was measured triple times and the mean value was recorded (with max. error 2.8%). Thermogravimetric Analysis (Perkin-Elmer, TGA 4000) was used to study the thermal decomposition kinetics of the samples under the following experimental conditions: (TG/DTG: 1–3 mg samples were examined at different heating rates of 1, 3, 5 and 7 K min^−1^ in the temperature range 30–500 °C under nitrogen flow of 20 mL min^−1^).

## Results and discussion

**Fig. 1:**
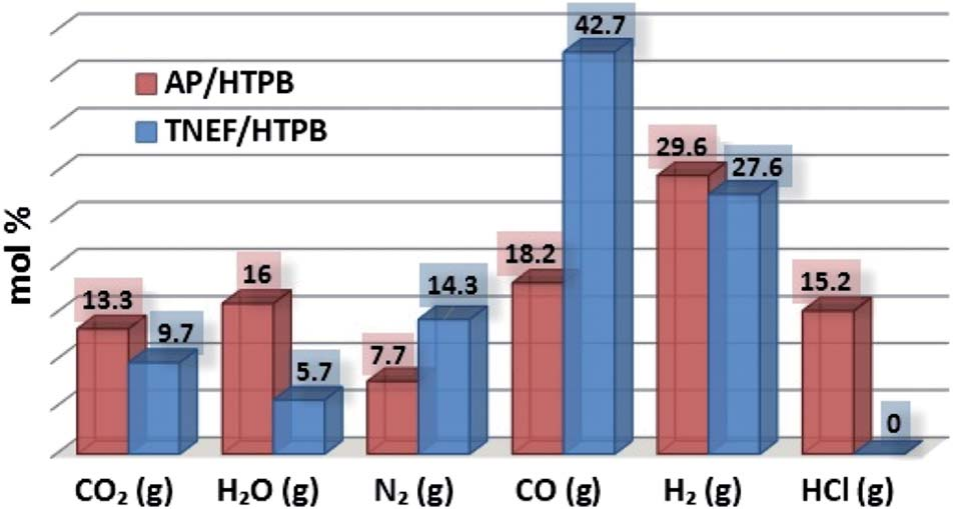
Reaction gaseous products at nozzle exit.

The characteristics calculations of TNEF as a new green high-energy dense oxidizer and TNEF/HTPB propellants formulation that have been tested by using EXPLO5 V_6.03 thermodynamic code showed interesting results comparing with that of AP and AP/HTPB. Fig. 1 shows the calculated mole percentage of reaction gaseous products at the nozzle exit for the most common AP/HTPB propellant formulation and the new green TNEF/HTPB propellant formulation. It is clear that AP/HTPB produce more than 15% toxic hydrochloric acid (HCl_(g)_) during the burning process. On the other hand, the new green propellant formulation TNEF/HTPB has no HCl_(g)_ in the burning gaseous products. In addition, TNEF has specific impulse (*I*_s_ = 250.1 s) and characteristic exhaust velocity (C* = 1408 m s^−1^) which are higher than that of AP (*I*_s_ = 156.9 s) and (C* = 947 m s^−1^). Moreover the new green propellant formulation TNEF/HTPB has also higher values of specific impulse (*I*_s_ = 231.5 s) and characteristic exhaust velocity (C* = 1425 m s^−1^) than the values of AP/HTPB (*I*_s_ = 228.2 s) and (C* = 1404 m s^−1^) respectively.

**Fig. 2:**
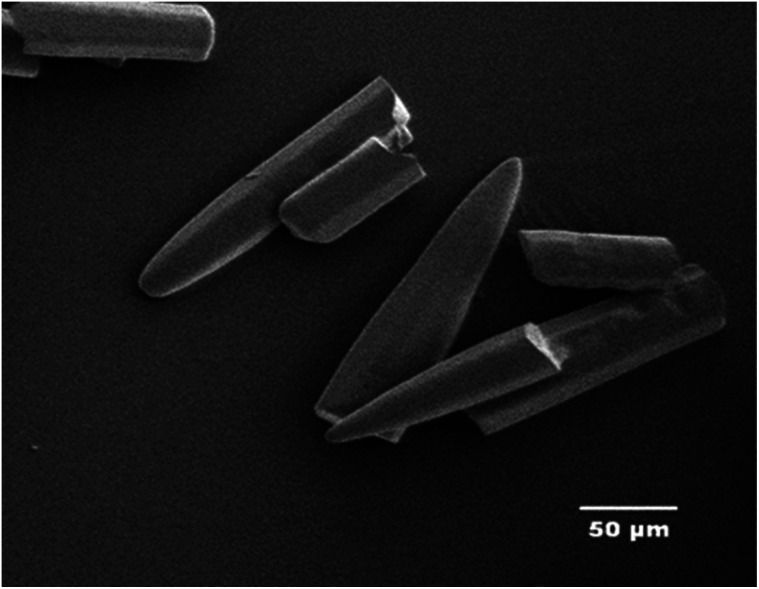
SEM of 2,2,2-trinitroethyl-formate.

TNEF was prepared as discussed in the experimental part, with a yield of 74%. The obtained crystals were colorless and SEM was used to study its crystal morphology. Hexagonal rods crystals with sharp edges were observed having approximate dimension of 70–200 μm length and 30 μm thickness as shown in Fig. 2. The crystals have smooth surface without cracks, while the sharp edges might affect the sensitivity characteristics of TNEF. The impact sensitivity was measured and found to be 5.4 J (50% probability of initiation) which is slightly higher than the traditional explosive RDX, while the friction sensitivity was 106 N.

**Fig. 3:**
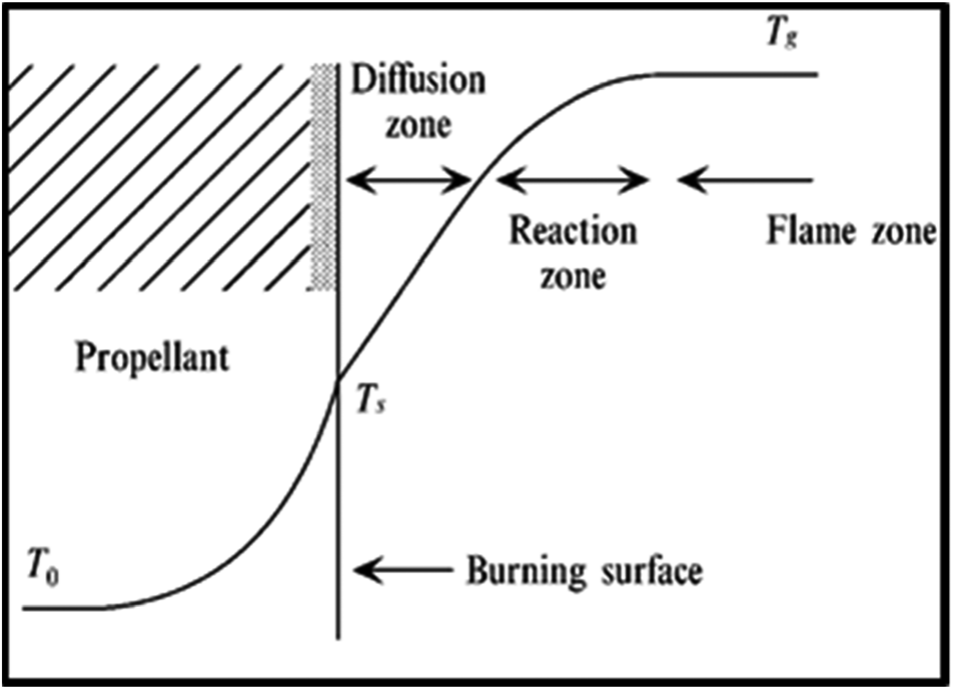
Combustion wave structure of an AP composite propellant.^41^

Results of the elemental analysis of TNEF in addition to the NMR spectroscopy and IR spectra are presented in the ESI.† According to the combustion theory,^41^ the decomposed gases diffusion process of the AP particles and the surrounding HTPB at burning surface controls the combustion mode of AP/HTPB composite propellants.^42–45^ The decomposed gases from the decomposition process of the AP particles and the HTPB binder react to produce heat on and above the burning surface. The HTPB binder as a fuel and the AP particles as an oxidizer diffuse and mix above the burning surface to form diffusional premixed flame (diffusion zone) and produce final combustion products such as CO_2_, H_2_O, N_2_, CO, H_2_ and HCl. The conductive heat feedback from the burning surface increase the temperature in the condensed phase from the initial propellant temperature (*T*_0_) to the burning surface temperature (*T*_s_). Then, increasing of the temperature occurs in the gas phase due to the exothermic reaction over the burning surface and reaches the final combustion temperature (*T*_g_) (see Fig. 3).

**Fig. 4:**
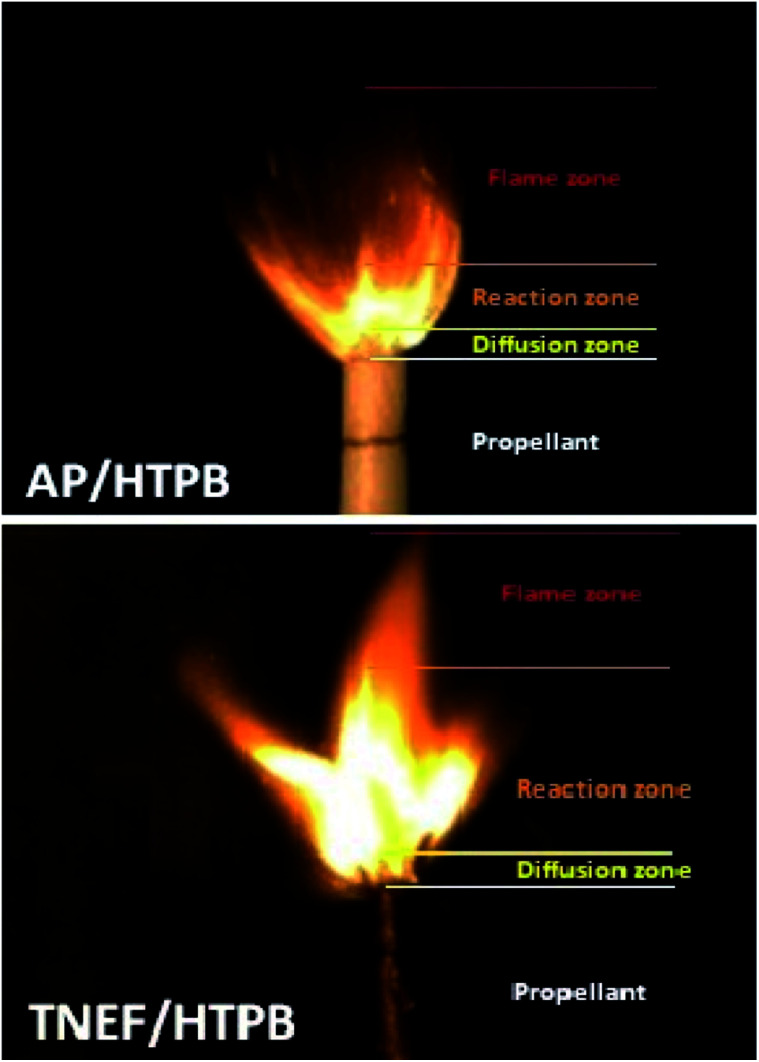
Burning behavior of the propellant samples.

The burning of the prepared samples (TNEF/HTPB and AP/HTPB) showed a uniform cigarette burning as shown in Fig. 4. The combustion process of the studied samples are controlled by the diffusion process of the decomposed gases of the oxidizer particles and the surrounding binder at the burning surface of the propellant. This zone (diffusion zone) is just above the burning surface and it seems to be dark where a series of degradation reactions occurs rapidly. It is clear that the diffusion zones of the two propellant formulations have almost the same thickness, which indicate that the new oxidizer (TNEF) is also diffuse in the HTPB matrix during the first burning stage of the new propellant formula. The decomposed gases reacted (oxidation reaction occurred) and produced heat above the burning surface. This zone is the reaction zone where a highly illuminated zone appeared as shown in Fig. 4. The thickness of the reaction zone of the new propellant formula is more than twice that of the traditional propellant formula and the intensity of brightness is higher. It means that the reaction between the fuel HTPB and the oxidizer TNEF is vigorous reaction. This may clarify the higher performance characteristics of the new propellant formulation than that of the traditional one. The final combustion products are formed above the reaction zone where thermal equilibrium of the combustion products happened, and this zone is known by the flame zone. The thickness of the flame zone of AP/HTPB propellant is more than that of the TNEF/HTPB. This result might be due to the high amount of gaseous products produced over the reaction zone during the combustion of AP/HTPB compared with that of TNEF/HTPB propellant in addition to the presence of HCl_(g)_ as a main gaseous product in case of the AP/HTPB burning which increase the flame zone with smoke of HCL_(g)_.

**Fig. 5:**
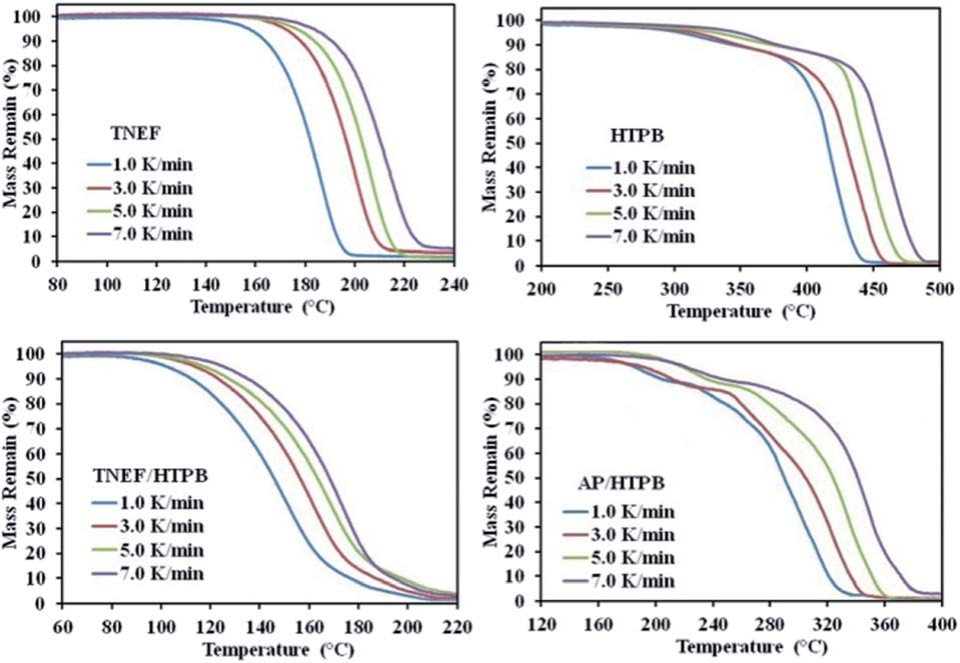
TGA thermograms of the studied samples.

The burning rate of TNEF/HTPB and AP/HTPB was measured by using high-speed camera as discussed in the experimental part. It was found that the burning rate of AP/HTPB propellant is 2.70 mm s^−1^, while the burning rate of the new propellant formulation TNEF/HTPB is 2.86 mm s^−1^. These results proved that TNEF/HTPB is a promising propellant formulation, which has higher burning rate than the traditional propellant AP/HTPB and the calculated burning characteristics are also higher. As a result, the decomposition kinetics of the two formulations was studied using thermal analysis technique. TG curves of TNEF, HTPB, TNEF/HTPB and AP/HTPB under four different heating rates 1, 3, 5, and 7 K min^−1^ were presented in Fig. 5. It is shown that a single decomposition step has been observed for TNEF that starts at 188.8 °C (onset temperature) and ends at 217.8 °C (onset temperature at the end of decomposition peak) in case of 5 K min^−1^ heating rate. HTPB decomposes on two stages starts at 330.0 °C for the first stage with mass loss ratio of 15% and at 441.3 °C for the second decomposition stage with final mass loss of about 97%, which means that HTPB can almost decompose to gaseous products completely. The new green propellants formulation showed a controlled homogenous one thermal decomposition step starts at 169.5 °C in case of 5 K min^−1^ heating rate, which can be the slow decomposition of TNEF that release large amount of heat, which leads to accelerate the thermal decomposition process of HTPB.

In addition, produce few gaseous products that cannot be released quickly. The reaction between these entrapped gaseous products release large amount of heat, which leads to accelerate the thermal decomposition process. TG thermogram of the AP/HTPB propellants showed two thermal decomposition stages that starts at 194.1 °C for the first stage. In the second stage, many kinds of oxidizing gases are generated and the thermal decomposition process accelerates due to release of large amount of heat.

The characteristic parameters of the TG curves and DTG peaks of the studied samples are listed in Table 1, which shows that the onset decomposition temperature and the initial mass loss temperature of the samples increased with increasing the heating rates. It is also obvious that the thermal decomposition reaction process of the common propellant formula that based on AP is more complicated than that of the new propellant formula, which is based on the new green high-energy dense oxidizer. It is clear that the decomposition temperature of the new propellant is lower than the traditional propellant (AP/HTPB) at each studied heating rate. The thermal decomposition reaction kinetics of all the studied samples are discussed using the conventional Kissinger method, which is based on the shift of decomposition peak temperature by changing the heating rate. The activation energies for the samples were calculated from the slope of the straight line by plotting ln(*β*/*T*^2^) *versus* 1/*T* for the four selected heating rates by applying Kissinger equation (see ESI†). The activation energy of the new oxidizer TNEF was found to be 146.4 kJ mol^−1^, while for the TNEF/HTPB was 125.6 kJ mol^−1^. The traditional propellants sample AP/HTPB had two activation energies 72.1 kJ mol^−1^ and 103.9 kJ mol^−1^ for the first and second steps of reaction respectively, which were lower than that of the new green propellant formula. Although the simplicity of this method, but it has a disadvantage, which is the inability to determine the reaction steps or discuss the distinct activation energy for each fraction conversion (*α*).

**Table 1:** TG/DTG data of the TNEF, HTPB, TNEF/HTPB and AP/HTPB^a^

Material	*β* [K min^−1^]	TG curves		DTG peaks	
		*T*_o_ [°C]	Mass loss [%]	*T*_p_ [°C]	*T*_e_ [°C]
TNEF	1.0	169.0	98.79	186.6	196.1
	3.0	184.5	97.67	200.3	211.8
	5.0	188.8	99.54	206.2	217.8
	7.0	192.1	97.06	209.6	221.0
HTPB	1.0 (1^st^)	301.2	14.81	322.5	357.8
	1.0 (2^nd^)	417.5	84.29	423.6	437.6
	3.0 (1^st^)	319.3	15.22	342.1	379.4
	3.0 (2^nd^)	432.7	84.07	442.9	465.6
	5.0 (1^st^)	338.4	13.89	353.4	392.2
	5.0 (2^nd^)	436.5	84.83	455.2	477.9
	7.0 (1^st^)	346.2	14.56	361.3	408.6
	7.0 (2^nd^)	442.0	84.08	461.6	485.7
TNEF/HTPB	1.0	139.8	99.13	163.7	171.2
	3.0	160.9	98.65	175.4	184.5
	5.0	169.5	96.09	182.9	192.3
	7.0	176.0	97.36	188.3	196.8
AP/HTPB	1.0 (1^st^)	168.0	11.82	189.9	193.6
	1.0 (2^nd^)	289.6	85.81	298.0	323.9
	3.0 (1^st^)	185.7	14.06	211.8	230.5
	3.0 (2^nd^)	303.4	84.59	322.9	344.8
	5.0 (1^st^)	194.1	12.68	228.2	235.7
	5.0 (2^nd^)	309.7	86.52	335.1	361.4
	7.0 (1^st^)	208.3	11.13	237.0	245.2
	7.0 (2^nd^)	317.1	85.29	349.8	366.8

a *T*_o_: onset decomposition temperature; *T*_e_: onset temperature of the end decomposition; *T*_p_: the peak temperature of mass loss rate; mass loss: from initial temperature to end temperature of DTG peak, (1^st^) first decomposition peak, (2^nd^) second decomposition peak.

**Fig. 6:**
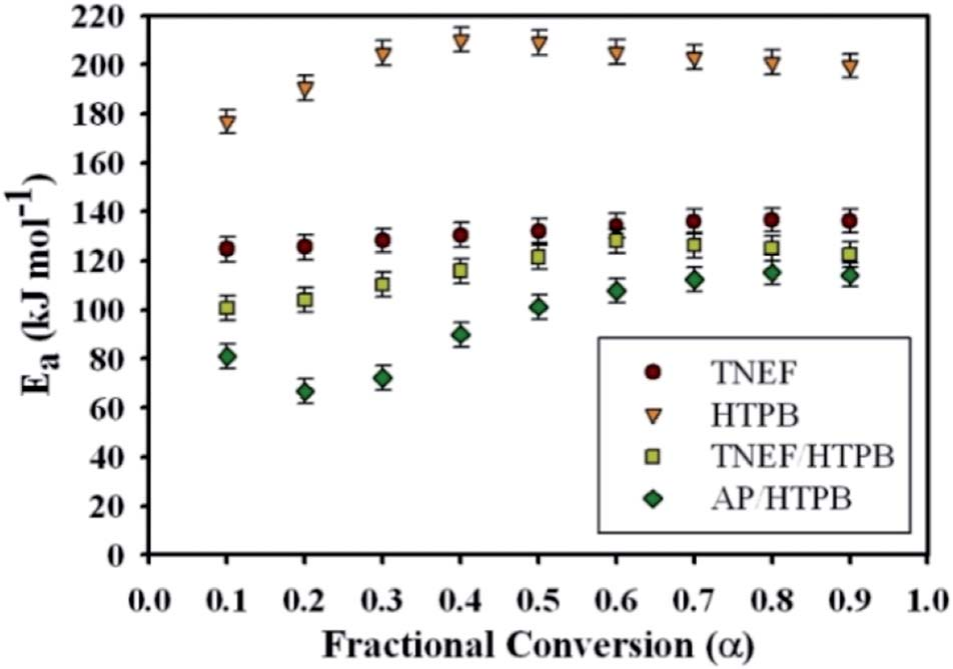
Activation energies at each conversion step (*α*) using OFW method.

Ozawa and Flynn–Wall developed an isoconversional calculation method which is commonly known as the OFW method to calculate the activation energy *E*_a_ through a plot of log *β versus* 1/*T* at each *α* regardless of the employed model using nonisothermal data (see ESI†).^46^ It was found that the activation energy of TNEF is varied from step to step of conversion with mean value of 132.1 kJ mol^−1^. The new green propellant formula TNEF/HTPB showed a higher value of activation energy than that of the traditional AP/HTPB propellant with mean value of 120.4 kJ mol^−1^. Fig. 6 shows the activation energy at each step of conversion *α* for TNEF, HTPB, TNEF/HTPB and AP/HTPB. The activation energy of the first stage thermal decomposition of AP/HTPB was 73.9 kJ mol^−1^, while for the second stage was 96.7 kJ mol^−1^. The activation energies of the fuel binder, HTPB, are higher than all the studied samples while its propellant based on the new oxidizer, TNEF, has the same behavior as the pure TNEF. The activation energies of the new propellant are higher than that of the traditional propellant (AP/HTPB). In order to confirm these results, another method named Kissinger–Akahira–Sunose (KAS) was also used to determine the activation energy at each degree of conversion using isoconversional method. Table 2 presents the kinetic data values of the studied samples using KAS method of calculation. By comparing the values using OFW method with those obtained by using the modified KAS method, a good agreement was detected. The activation energy of TNEF obtained by KAS equation (see ESI†) was found to be 131.5 kJ mol^−1^. The new propellant formula has a mean value of activation energy equal 119.4 kJ mol^−1^, which is higher than that of the traditional propellants formula AP/HTPB (92.4 kJ mol^−1^). As commonly suggested in literatures, the mean values of activation energy using OFW and KAS methods were calculated in the interval of (*α* = 0.3–0.7) due to the large influence of the experimental conditions specially in case of TG/DTG on the data quality of the process “tails”.^47–50^

**Table 2:** Kinetic data of TNEF, HTPB, TNEF/HTPB andAP/HTPB obtained using the modified KAS method

*α* reacted	TNEF			HTPB			TNEF/HTPB			AP/HTPB		
	*E*_a_	log *A*	*r*	*E*_a_	log *A*	*r*	*E*_a_	log *A*	*r*	*E*_a_	log *A*	*r*
0.1	123.8	12.08	0.9993	176.2	10.86	0.9933	99.3	10.35	0.9893	76.8	5.09	0.9694
0.2	124.7	11.98	0.9989	189.1	11.68	0.9991	102.6	10.33	0.9944	61.3	3.18	0.9744
0.3	127.3	12.15	0.9991	203.1	12.57	0.9954	109.0	10.82	0.9958	66.9	3.53	0.9802
0.4	129.6	12.31	0.9986	209.4	12.96	0.9985	114.8	11.28	0.9968	85.3	5.13	0.9781
0.5	131.6	12.45	0.9994	208.6	12.83	0.9992	120.8	11.78	0.9972	97.1	6.10	0.9874
0.6	133.6	12.60	0.9988	204.2	12.43	0.9987	127.3	12.30	0.9976	103.9	6.61	0.9865
0.7	135.4	12.72	0.9989	202.0	12.19	0.9993	125.2	12.01	0.9987	108.7	6.95	0.9909
0.8	136.0	12.71	0.9991	199.5	11.93	0.9989	123.4	11.67	0.9977	111.4	7.09	0.9934
0.9	135.5	12.55	0.9984	197.9	11.69	0.9981	119.6	11.43	0.9985	110.4	6.85	0.9952
Mean	131.5	12.45		205.5	12.60		119.4	11.64		92.4	5.66	

## Conclusions

2,2,2-Trinitroethyl-formate (TNEF) is a new interesting (chlorine-free) green high-energy dense oxidizer (HEDO), which can be easily prepared from 2,2,2-trinitroethanol (TNE). The burning characteristics calculated for the new green propellant formula based on TNEF with HTPB was higher than that of traditional propellant based on AP/HTPB. The TNEF/HTPB propellant does not produce any toxic HCl_(g)_ in the burning process that makes it environmentally safe comparing with the traditional propellant formula AP/HTPB which produce about 15% HCl_(g)_ (mol%). The measured burning rate of TNEF/HTPB (2.86 mm s^−1^) was higher than AP/HTPB (2.70 mm s^−1^). A uniform cigarette burning was observed for both of the studied propellant samples with nearly the same diffusion zone and higher intensity of brightness in case of TNEF/HTPB which is compatible with the calculated results. The kinetic study by the different three methods showed activation energy of TNEF in the range of 131–146 ± 0.5 kJ mol^−1^, while the activation energy of TNEF/HTPB propellant is in the range of 119–126 ± 0.4 kJ mol^−1^, which is higher than that of AP/HTPB (88–97 ± 0.3 kJ mol^−1^). The new TNEF/HTPB formulation is an interesting propellant composition which might be candidate to replace the toxic traditional composite solid rocket propellant AP/HTPB.

## Conflicts of interest

There are no conflicts to declare.

## Supplementary Material

RA-008-C8RA01515E-s001
