# Supplementary material for: Environmentally safe (chlorine-free): new green propellant formulation based on 2,2,2-trinitroethyl-formate and HTPB
Source: RSC Adv. 2018 Mar 27;8(21):11771–7. doi: 10.1039/c8ra01515e (PMC9079109; doi:10.1039/c8ra01515e)
Supplement: RA-008-C8RA01515E-s001 [file RA-008-C8RA01515E-s001.pdf]

Electronic Supplementary Material (ESI) for RSC Advances.
This journal is © The Royal Society of Chemistry 2018

Supplementary Information

1. TNEF Characterization

The new promising oxidizer (TNEF) was characterized by ^{1}H, ^{13}C, and ^{14}N NMR spectroscopy. The $CH_2C(NO_2)_3$ singlet moiety in the ^{1}H NMR spectra can be determined at δ = 5.53 ppm in $[D_6]$acetone. The methylene groups carbon resonances (CH_2) could be observed at 63.9 ppm, while the carbon atom of the formate group was identified at 113.3 ppm and the trinitromethyl groups $C(NO_2)_3$ at 125.5 ppm in the ^{13}C NMR spectra. The nitrogen atom of $C(NO_2)_3$ group was found at –33.3 ppm in ^{14}N NMR spectra. The elemental analysis showed good results for TNEF ($C_7H_7N_9O_{21}$, $M_{wt.}$=553.18 g mol^{-1}) that found (C 15.11, H 1.34, N 21.56 %), which are quite near to the calculated values (C 15.20, H 1.28, N 22.79 %).

The vibrational analysis (IR spectra) of TNEF showed the characteristic asymmetric NO_2 stretching vibrations at 1585 (vs) cm^{-1}. The C–H stretching vibrations were found at 2957 (w) cm^{-1} and the CNO_2 at 855 (w) cm^{-1}, where, (vs = very strong), and (w = weak).

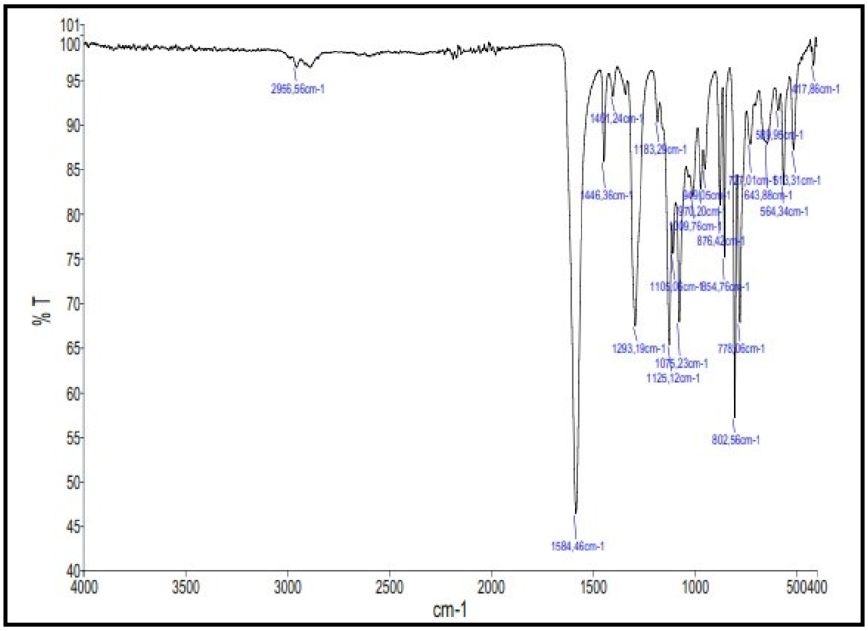

Fig. 1 FTIR of 2,2,2trinitroethyl-formate (TNEF)

2. Kinetic analysis

The kinetic triplets which are the activation energy (E_a), pre-exponential factor (A) and kinetic model ($f(\alpha)$) represent the kinetic analysis. For complete description of the kinetics, these three kinetic parameters should be determined for each reaction step. Nowadays, a large number of analytical methods that can be used to determine the kinetic parameters of distinct solid-phase reactions evaluation are available. The kinetic parameters can be determined either isothermally or nonisothermally by using two main methods which are: Isoconversional (model-free) and model-fitting methods.

Calculation of the activation energy:

The activation energy (E_a) of the exothermic decomposition reaction of the prepared propellants samples can be calculated by easy way using Kissinger's method.

$$-\frac{Ea}{R} = \frac{d\ln(\beta/T_P^2)}{d(1/T_P)}$$

Where β is the heating rate and T_p is the DTG peak temperature at that rate. By plotting of $\ln(\beta/T_p^2)$ versus $1/T_p$, the activation energy can be calculated from the slope of this straight line. Such rough temperature integral approximation may cause an inaccurate calculated values of E_a.[1] More accurate equation was presented according to Starink [2] for E_a calculation which is commonly called the Kissinger–Akahira–Sunose (KAS) equation:[3]

$$\ln\left(\frac{\beta_i}{T_{\alpha,i}^{1.92}}\right) = const - 1.0008\frac{E_\alpha}{RT_\alpha}$$

Ozawa and Flynn–Wall (OFW) used a nonisothermal data and developed an isoconversional calculation method (commonly referred to as the OFW method), in which taking the logarithm of the nonisothermal rate law to give the following equation:[4]

$$\log g(\alpha) = log\frac{AE_a}{\beta R} + log\int_{\frac{E_a}{RT}}^{\infty}\frac{e^{\frac{-E_a}{RT}}}{\left(\frac{E_a}{RT}\right)^2}d\frac{E_a}{RT}$$

By substitution using Doyle's approximation:[5-7]

$$log\int_{\frac{E_a}{RT}}^{\infty}\frac{e^{\frac{-E_a}{RT}}}{\left(\frac{E_a}{RT}\right)^2}d\frac{E_a}{RT} \approx -2.315 - 0.4567\frac{E_a}{RT}$$

Then OFW equation can be written as:

$$\log\beta = log\frac{AE_a}{g(\alpha)R} - 2.315 - 0.457\frac{E_a}{RT}$$

Ref.:

1 S. Vyazovkin, A. K. Burnham, J. M. Criado, L. A. Pérez-Maqueda, C. Popescu and N. Sbirrazzuoli, *Thermochimica acta*, 2011, **520**, 1-19.

2 M. Starink, *Thermochimica Acta*, 2003, **404**, 163-176.

3 T. S. T. Akahira, *Res. Report Chiba Inst. Technol. (Sci. Technol.)*, 1971, **16**, 22-31.

4 D. D. Sergey Vyazovkin, *Journal of chemical information and computer sciences*, 1996, **36**, 42-45.

5 C. Doyle, *Appl Polym Sci* 1961, **5**, 285–292.

6 D. C. CD Doyle, *Nature*, 1965, 1-12.

7 C. Doyle, *Appl Polym Sci*, 1962, **6**, 639–642.
